# 非小细胞肺癌新辅助免疫治疗的研究进展、获益人群、治疗周期和疗效预测

**DOI:** 10.3779/j.issn.1009-3419.2022.101.01

**Published:** 2022-02-20

**Authors:** 敬斌 纪, 宸瑜 张, 垒 彭, 文捷 矫

**Affiliations:** 266071 青岛，青岛大学附属医院胸外科 Department of Thoracic Surgery, Affiliated Hospital of Qingdao University, Qingdao 266071, China

**Keywords:** 肺肿瘤, 围术期, 免疫治疗, 新辅助治疗, 免疫检查点抑制剂, 综述, Lung neoplasms, Perioperative, Immunotherapy, Neoadjuvant therapy, Immune checkpoint inhibitors, Review

## Abstract

免疫检查点抑制剂（immune checkpoint inhibitors, ICIs）的出现极大程度上改变了非小细胞肺癌（non-small cell lung cancer, NSCLC）患者的治疗前景，术前新辅助免疫治疗方案作为一种有效、安全的治疗方案逐渐受到重视。然而，新辅助免疫治疗方案相关的研究起步时间较晚，研究成果相对较少且主要集中于样本量较小的Ⅰ期、Ⅱ期研究，治疗方案本身存在也许多尚不明确的地方，在获益人群的筛选、治疗周期的选择和疗效预测等方面也尚未达成广泛的共识。本文综述了与新辅助免疫治疗相关的重要研究和新近成果，旨在以这些研究的成果为基础，从获益人群、治疗周期和疗效预测三个方面全方位探讨该类治疗的流程及存在的问题。

## Abstract (en)

The emergence of immune checkpoint inhibitors (ICIs) has dramatically changed the therapeutic outlook for patients with non-small cell lung cancer (NSCLC). Preoperative neoadjuvant immunotherapy has been paid more and more attention as an effective and safe treatment. Neoadjuvant immune therapy, however, the relevant research started late, relatively few research results and mainly focused on the small sample size of phase Ⅰ and Ⅱ studies, treatment itself exists many places it is not clear, also in benefit population screening, the respect such as the choice of treatment and curative effect prediction has not yet reached broad consensus. This paper reviews the important studies and recent achievements related to neoadjuvant immunotherapy, aiming to comprehensively discuss the procedures and existing problems of this kind of therapy from three aspects of beneficiary groups, treatment cycle and efficacy prediction.

肺癌是全球范围内第二常见且死亡率最高的癌症，主要类型分为非小细胞肺癌（non-small cell lung cancer, NSCLC）和小细胞肺癌（small cell lung cancer, SCLC）。在2020年有近180万人死于肺癌，其中NSCLC约占85%^[1-3]^。根据2021年的美国国立综合癌症网络（National Comprehensive Cancer Network, NCCN）指南，手术仍是治疗可切除的Ia期-Ⅲa期NSCLC肺癌最常用、最有效的治疗方式^[4]^。20世纪90年代以来，多项研究证实了术前新辅助化疗、术后辅助化疗均较单纯手术可提高患者的总体生存时间（overall survival, OS）和无进展生存期（progression-free survival, PFS）^[5-11]^，但因其较为明显的毒副作用导致患者耐受性较差，5年生存率仅提升5%^[9-11]^。对大量可行手术切除的NSCLC患者，寻找耐受性好、可进一步提升患者生存期和生存率的新辅助治疗和辅助治疗方案已经成为一项重要的研究方向。

近年来，通过阻断程序性细胞死亡蛋白-1（programmed cell death protein 1, PD-1)/程序性死亡配体-1（programmed cell death-ligand 1, PD-L1）和细胞毒性T淋巴细胞相关蛋白4（cytotoxic T lymphocyte-associated antigen-4, CTLA-4）通路与抑制性抗体的相互作用，使活化的T细胞释放细胞因子、穿孔物、颗粒酶等来杀伤肿瘤细胞的免疫治疗方案迅速发展。运用免疫检查点抑制剂（immune checkpoint inhibitors, ICIs）术前新辅助免疫治疗和术后辅助免疫治疗方案也被作为术前新辅助化疗和术后辅助化疗方案的替代方案加以研究。目前，已有大量Ⅲ期临床研究证明，术后辅助免疫治疗在耐受性、安全性、疗效等众多方面均较术后辅助化疗方案显示出优势^[12-19]^。然而，目前对术前新辅助免疫治疗的研究较少，且主要集中于Ⅰ期、Ⅱ期研究。从机制上说，PD-1/PD-L1抑制剂杀死肿瘤时，抗原提呈细胞（antigen-presenting cell, APC）被宿主T细胞所识别。当肿瘤体积相对较大时，APC的抗原负荷相对较大，从而可引起较强的抗肿瘤T细胞反应。因此，术前应用的新辅助免疫治疗的疗效理论上优于术后应用的辅助免疫治疗，并可能产生更大的长期生存效益^[20]^。然而，由于新辅助治疗的治疗时间为术前，不可避免的问题是实行新辅助治疗一定程度上会推迟进行手术治疗的时间，可能导致肿瘤进一步扩散，对患者手术治疗的疗效产生影响。因此，明确新辅助免疫治疗的获益人群、制定合适的新辅助免疫治疗方案和该疗法疗效进行预测成为临床上应用此类型治疗方案的主要问题。本文综述了NSCLC新辅助免疫治疗相关的多项研究成果和最新的研究进展，旨在从获益人群的选择、治疗方案的制定和对该疗法疗效进行预测等方面，全面探讨此类治疗的全过程。

## 1 NSCLC新辅助免疫治疗研究进展

目前，已有大量Ⅰ期、Ⅱ期研究结果显示，新辅助免疫治疗可在NSCLC的综合治疗中发挥重要作用。在已发布的《非小细胞肺癌新辅助免疫治疗国际专家共识》中，可切除的Ib期-Ⅲa期NSCLC患者可考虑使用新辅助免疫治疗联合含铂的双药化疗或新辅助单药免疫治疗。在Ⅲ期临床研究上，目前有多种单药及联合方案新辅助免疫治疗的Ⅲ期实验正在进行中，但尚未有研究报告发表，且除CheckMate-816研究外未公布研究结果和数据。

### 1.1 新辅助免疫治疗研究临床终点的选择

选择合适的临床终点是新辅助免疫治疗相关研究的重点问题。在新辅助和辅助治疗的临床试验中，主要终点的金标准是OS。但由于获得最终的OS结果需要较长时间的随访研究，且目前大部分Ⅲ期研究均在进行中且尚未取得研究成果，该类型研究普遍选择病理完全缓解率（pathological complete response, pCR）、主要病理缓解率（major pathologic response, MPR）和无事件生存期（event free survival, EFS）作为临床终点。pCR定义为在手术切除时没有任何可行的肿瘤，MPR定义为小于10%的存活肿瘤，二者均已被证明和肺癌死亡风险密切相关^[21-23]^。目前的实验数据^[23]^显示，较高的MPR、pCR与较高的手术切除率和较高的OS相关。然而，pCR和MPR缺乏标准化和前瞻性数据，波动范围较大。在Ⅲ期临床研究中，已获得部分结果的CheckMate-816实验选择了pCR和EFS作为主要临床研究终点，其他研究的主要临床终点通常在pCR、OS和EFS中选择，且大多数研究均将MPR作为次要研究终点。值得注意的是，有相当部分的Ⅲ期试验最初将MPR定为主要研究终点，后将pCR改为主要研究终点，这可能说明pCR作为该类研究的临床终点较MPR有更大的优势。

由于目前大部分研究进行随访研究的时间尚较短且新辅助免疫治疗普遍要求术后由病理医生评估病理缓解情况，pCR、MPR和EFS仍是最受重视的临床终点，且pCR可能略优于MPR。随着Ⅲ期临床研究逐步发表结果及随访数据的进一步完善，OS可能在一段时间后会成为最重要的研究终点，且MPR和pCR的标准可能会被进一步标准化制定。

### 1.2 新辅助免疫治疗Ⅰ期、Ⅱ期研究

Ⅰ期、Ⅱ期研究通常选择MPR和pCR作为有效性的研究终点，选择3级及以上治疗相关不良事件（treatment-related adverse event, TRAEs）发生率作为安全性的研究终点。同时，有部分研究选择使用世界卫生组织制定的实体瘤疗效评价标准（Response Evaluation Criteria In Solid Tumors, RECIST）对方案进行综合评估。

#### 1.2.1 新辅助单药免疫治疗

新辅助单药免疫治疗在PD-1/PD-L1抑制剂应用上最先取得了研究进展。目前，美国食品药品监督管理局（Food and Drug Administration, FDA）批准应用于NSCLC治疗的四种PD-1/PD-L1抑制剂分别为PD-1抑制剂：纳武利尤单抗（Nivolumab）、帕博利珠单抗（Pembrolizumab）以及PD-L1抑制剂：阿替利珠单抗（Atezolizumab）、度伐利尤单抗（Durvalumab）。由于度伐利尤单抗的用药对象为Ⅱ期/Ⅲ期不可切除的NSCLC，不用于术前新辅助治疗。其他3种药物均有已发表的研究结果证实应用于新辅助免疫治疗NSCLC的价值。此外，国产的PD-1抑制剂信迪利单抗（Sintilimab）亦取得了较好的实验成果，已于2021年6月获得国家药品监督管理局的批准，联合化疗应用于晚期鳞状NSCLC的一线治疗。

2018年发表的前瞻性Ⅱ期临床研究Checkmate-159是最早开展的NSCLC新辅助免疫治疗研究。该研究入组21例初治的Ia期-Ⅲa期可切除NSCLC患者，术前接受2个周期纳武利尤单抗（3 mg/kg，2周1次）的新辅助免疫治疗方案，于第4周进行手术。20例患者达到了R0切除，MPR和pCR分别达45%和15%，远高于过去新辅助化疗方案的20%和4%^[9-11]^。在随访过程中，该方案18个月无复发生存率达73%。该研究3级及以上TRAEs发生率为4.5%。按照RECIST进行评估后认为，该方案副作用小、患者耐受性好且不会延迟应用手术治疗的时间。该研究首次显示了新辅助免疫疗法的有效性和安全性。该项研究取得的MPR（45%）高于其他免疫单药新辅助治疗的结果，最大的缺陷是样本量较小，单臂实验无对照组等^[24]^。

Ⅱ期研究LCMC3是对新辅助免疫治疗样本量最大的研究，纳入来自美国15个医疗中心的180例Ⅰb期-Ⅲa期及部分Ⅲb期（T3N2）可切除NSCLC患者。每名患者使用2个周期的阿替利珠单抗（1200 mg，3周1次）治疗后手术，旨在评估该方案的安全性和有效性。2019年更新的研究结果显示，在Ⅰb期-Ⅲa期及部分Ⅲb期（T3N2）患者中使用两个周期的阿替利珠单抗新辅助治疗方案MPR达19%（15/77），pCR达5%（4/77），3级及以上TRAEs发生率为10%。按照RECIST标准进行评估后，认为该方案安全性良好、耐受性好。此外，该实验的MPR被证实与PD-L1表达水平无关，PD-L1或对该类方案预测效果较差^[25, 26]^。2021年更新的数据^[27]^分析显示，2个周期的阿替利珠单抗治疗方案MPR达20.4%，pCR达6.8%，均远高于过去新辅助化疗所取得的数据。在安全性上，3级及以上TRAEs发生率为16%。由于该研究的缺陷在于样本来自多个医疗中心且没有对照组，可能会因为各医疗中心的医疗条件差异而产生误差。

Ⅱ期研究TOP1501对32例Ⅰb期-Ⅲa期的NSCLC患者采用了术前接受2个周期帕博利珠单抗（200 mg，3周1次）新辅助免疫治疗。研究结果显示在该方案最终切除的25例患者中MPR达28%，且病理缓解≥50%的患者达80%。术后最常见的不良事件是心房颤动（24%）^[28]^。

ChiCTR-OIC-17013726研究是一项由中国医学肿瘤科学院牵头开展的Ⅰ期研究，纳入了31例Ⅰa期-Ⅲb（T3N2）期可切除NSCLC鳞状细胞癌患者，术前进行两次信迪利单抗给药（200 mg，3周1次），主要研究终点是MPR和客观有效率（objective response rate, ORR）。研究^[29]^结果显示，信迪利单抗单药新辅助免疫MPR率40.5%，pCR为16.2%，3级及以上TRAEs发生率为10%。这一结果证明信迪利单抗用于术前新辅助治疗Ⅰa期-Ⅲb期可切除NSCLC鳞状细胞癌患者安全有效。在2021年美国临床肿瘤学会（American Society of Clinical Oncology, ASCO）大会上，该研究团队公布的最新数据显示该方案1年DFS达91.7%，2年DFS和OS分别达73.3%和87.5%。

根据上述已发表研究结果的Ⅰ期、Ⅱ期单臂研究（表 1），可以认为对可切除的Ⅰb期-Ⅲa期NSCLC患者（排除*EGFR*突变及*ALK*重排）新辅助单药免疫治疗方案无论应用PD-1抑制剂纳武利尤单抗、帕博利珠单抗，还是PD-L1抑制剂阿替利珠单抗，在保证安全性的前提下取得较高的MPR（19%-45%）。由于上述研究均为单臂研究，缺少对照组，无法直观地与化疗等其他治疗方案进行对比，但与过去新辅助化疗相关研究（MPR约20%，pCR约4%）相比，MPR和pCR有明显改善。新辅助单药免疫治疗方案是可行有效的。由于目前取得成果的Ⅱ期研究样本量均较小，尚无法在几种药物中进行方案优选，可能需进一步研究确定。

**表 1:** 新辅助ICIs单药治疗Ⅰ期、Ⅱ期试验结果 Results of phase Ⅰ and Ⅱ trials of neoadjuvant ICIs monotherapy

Registration No.	Study name	Phase	Intervention	*N*	Stage	ICIs doses per cycle	Frequency	No. of cycles	TRAEs (≥G3) (%)	MPR (%)	pCR (%)	Survival
NCT02259621	CheckMate-159	Ⅱ	Nivolumab (single arm)	21	Ⅰa-Ⅲa	3 mg/kg	*q2w*	2	4.5	45	15	18-month RFS: 73%
NCT02927301	LCMC3	Ⅱ	Atezolizumab (single arm)	180	Ⅰb-Ⅲa, Ⅲb (T3N2)	1, 200 mg	*q3w*	2	16	21	7	1-year DFS: 85%; 1-year OS: 95%
NCT02818920	TOP1501	Ⅱ	Pembrolizumab (single arm)	25	Ⅰb-Ⅲa	200 mg	*q3w*	2	6	28	No data	No data
ChiCTR-OIC-17013726		Ⅰ	Sintilimab (single arm)	31	Ⅰb-Ⅲa	200 mg	*q3w*	2	10	40.5	16.2	1-year DFS: 91.7%; 2-year DFS: 73.3%; 2-year OS: 91.7%
ICIs: immune checkpoint inhibirtors; TRAE: treatment-related adverse event; MPR: major pathologic response; pCR: pathological complete response; RFS: recurrence-free survival; OS: overall survival; DFS: disease-free survival.												

#### 1.2.2 新辅助免疫治疗联合化疗

在免疫治疗取得良好的进展后，许多研究开始关注于免疫治疗联合化疗或其他药物的联合治疗方案。在术后辅助免疫治疗方面，已有多项Ⅲ期研究支持术后ICIs联合化疗这类治疗方案。KEYNOTE-189、KEYNOTE-407这两项双盲3期临床实验表明无*EGFR*或*ALK*突变的转移性NSCLC初治患者，帕博利珠单抗联合化疗的疗效均优于化疗（OS、PFS均显著改善）^[30, 31]^。IMpower130双盲3期临床实验表明在Ⅳ期非鳞状NSCLC患者中应用阿替利珠单抗联合化疗的疗效优于化疗^[32]^。NCCN指南基于上述实验结果，认定帕博利珠单抗联合卡铂+培美曲塞或卡铂加/不加紫杉醇为转移性NSCLC（无*EGFR*/*ALK*突变）的一线治疗方案；认定阿替利珠单抗用于已接受靶向治疗且在含铂化疗期间/之后病情进展的转移性NSCLC患者。

在新辅助免疫治疗方面，目前已有多项Ⅱ期试验证明在Ⅰb期-Ⅲa期NSCLC患者中应用术前新辅助免疫治疗联合化疗这一方案的有效性。Ⅱ期研究NADIM试验将46例可切除的Ⅲa期NSCLC患者在术前接受3个周期纳武利尤单抗联合紫杉醇+卡铂，结果显示在有效性上，41例最终接受手术的患者MPR为83%（34/41），pCR为63%（26/41），90%（37/41）的患者实现了临床分期的病理降级；在安全性上，30%的患者发生3级及以上TRAEs发生率，最常见的3级及以上TRAEs是脂肪酶升高（7%）和发热性中性粒细胞减少（7%），但没有不良事件与手术延迟或死亡相关^[33]^。Ⅱ期研究NCT02716038纳入30例Ib期-Ⅲa期NSCLC患者，术前接受4个周期阿替利珠单抗联合卡铂和白蛋白结合型紫杉醇，结果显示在有效性上，26例成功切除的患者MPR达50%，pCR达21.4%；最常见的3级及以上TRAEs是中性粒细胞减少（50%）、丙氨酸转氨酶升高（7%, 2/30），天冬氨酸转氨酶升高（7%）、血小板减少2例（7%），没有与治疗有关的死亡^[34]^。

上述实验显示，在新辅助单药免疫治疗的基础上结合含铂的双药化疗方案组成的新辅助联合治疗方案对Ib期-Ⅲa期可切除NSCLC患者是有效的术前治疗方案，在OS、MPR和pCR等方面获益更加明显。尽管相较于新辅助免疫单药治疗方案3级及以上TRAEs发生率有所上升，仍在可耐受范围内。除上述Ⅱ期研究外，Ⅲ期研究CheckMate-816已证明纳武利尤单抗联合含铂的双药化疗这一新辅助免疫治疗方案的有效性，该研究将于后文详细阐述。

#### 1.2.3 双ICIs（O+Y）新辅助免疫治疗方案

伊匹单抗（Ipilimumab）是一种单克隆抗体，能与CTLA-4结合，阻碍CTLA-4与配体的相互作用，增加T细胞的活化和增殖，从而达到抗肿瘤治疗的目的。基于CheckMate-9LA、Hellmann MD和CheckMate 568等实验，纳武利尤单抗联合伊匹单抗（O+Y）这一双ICIs治疗方案被认可为PD-L1≥1%的NSCLC患者的一线治疗方案^[35-37]^。许多研究者尝试PD-1/PD-L1抑制剂联合CTLA-4单抗是否可以同样用于新辅助免疫治疗，这类研究取得了一些进展。

Ⅱ期试验NEOSTAR对44例可切除的Ⅰ期-Ⅲa期NSCLC患者中的23例患者行纳武利尤单抗（O药）单药治疗，同时对剩余的21例患者行纳武利尤单抗联合伊匹单抗（O+Y药方案），主要研究终点为MPR。研究结果表明，总体MPR率为25%，pCR率为18%；O+Y联合用药组的MPR、pCR均高于单药治疗组，分别为MPR：50% *vs* 24%；pCR：38% *vs* 10%。后续对安全性的研究发现O+Y方案3级及以上TRAEs发生率较O药单药治疗无显著差异（10% *vs* 13%），也未增加围手术期发病率及死亡率。因此可以认为该实验证实了对可切除的Ⅰ期-Ⅲa期NSCLC患者，纳武利尤单抗联合伊匹单抗新辅助治疗方案较纳武利尤单抗单药方案可以在保证安全性的同时使患者获益^[38]^。

### 1.3 新辅助免疫治疗Ⅲ期临床研究

新辅助免疫治疗的Ⅲ期试验主要聚焦于FDA批准应用于NSCLC治疗的四种PD-1/PD-L1抑制剂，此外国产PD-1抑制剂替雷利珠单抗（Tislelizumab）也有Ⅲ期试验在进行。目前，正在进行的新辅助免疫治疗Ⅲ期临床研究包括CheckMate-816（NCT02998528，纳武利尤单抗+含铂双药物化疗）^[39]^、IMpower030（NCT03456063，阿替利珠单抗+含铂药物化疗）^[40]^、KEYNOTE-671（NCT03425643，帕博利珠单抗+含铂双药化疗）^[41]^、AEGEAN（NCT03800134，度伐利尤单抗+含铂药物化疗）^[42]^、CA209-77T(NCT04025879，纳武利尤单抗+含铂双药化疗）和NCT04379635（替雷利珠单抗+含铂双药化疗）等（表 2）。截至2021年11月，已有一项Ⅲ期研究（CheckMate-816）取得相关的研究成果。

**表 2:** 新辅助免疫治疗Ⅲ期临床研究 Phase Ⅲ clinical study of neoadjuvant immunotherapy

Registration No.	Study name	Phase	*N* (Plan)	Start date	Patients with stage	Neoadjuvant therapy group	ICIs doses per cycle	Frequ-ency	No. of cycles	Control group	Primary outcome measures	Status
NCT02998528	Checkmate-816	Ⅲ	358	2017	Ⅰb-Ⅲa	Nivolumab+PT-DC	360 mg	*q3w*	3	PT-DC	pCR, EFS	On going
NCT03456063	IMpower 030	Ⅲ	453	2018	Ⅱa-Ⅲa, Ⅲb (T3N2)	Atezolizumab +PT-C	1, 200 mg	*q3w*	4 (Postop -erative: 16)	Placebo+PT-DC	EFS	On going
NCT03425643	MK-3475-671/KEYNO-TE671	Ⅲ	786	2018	Ⅱa-Ⅲa, Ⅲb (T3/T4N2)	Pembrolizumab+PT-DC	200 mg	*q3w*	4	Placebo+PT-DC	OS, EFS	On going
NCT03800134	AEGEAN	Ⅲ	800	2018	Ⅱa-Ⅲa, Ⅲb (N2)	Durvalumab +PT-C	1, 500 mg	*q3w*	4	Placebo+PT-DC	pCR, EFS	On going
NCT04025879	CA209-77T	Ⅲ	452	2019	Ⅱa-Ⅲa, Ⅲb (T3N2)	Nivolumab+PT-DC	No data	No data	No data	Placebo+PT-DC	EFS	On going
NCT04379635		Ⅲ	380	2020	Ⅱa-Ⅲa	Tislelizumab+PT-DC	200 mg/400 mg	*q3w*/*q6w*	12	Placebo+DDP/CBP+PTX/MTA	MPR, EFS	On going
EFS: event free survival; PT-C: platinum-based chemotherapy; PT-DC: platinum-based doublet chemotherapy.												

CheckMate-816是一项随机、开放标签、多中心的Ⅲ期临床研究，共纳入358例Ib期-Ⅲa期NSCLC患者，术前随机接受纳武利尤单抗（360 mg）联合含铂的双药化疗或单用含铂的双药化疗（3周1次，最多3个周期），分为新辅助免疫联合化疗组和化疗组。主要研究终点是两组的pCR和EFS，关键次要终点是OS、MPR和至死亡或远处转移的时间^[39]^。

2021年4月该研究公布的部分结果显示联合治疗组的MPR和pCR明显高于化疗组，分别为36.9% *vs* 8.9%（*P* < 0.000, 1）和24% *vs* 2.2%（*P* < 0.000, 1）。同时，安全性相关的评估结果显示，两个治疗组的患者接受手术（83% *vs* 75%）和实现肿瘤完全切除（83% *vs* 78%）的比例并无显著差异，手术相关不良事件的发生率相似（3级及以上TRAEs发生率：19% *vs* 21%），这表明在安全性上联合治疗方案较单纯化疗没有明显差异^[43, 44]^。2021年11月该研究的研究团队宣布，相较于术前单用化疗的患者，术前接受纳武利尤单抗联合化疗方案的患者的EFS显示出具有统计学意义和临床意义的改善。至此，CheckMate-816研究达到了两个主要研究终点pCR和EFS，二者均有统计学意义上的改善。上述研究结果表明，纳武利尤单抗联合化疗这一方案较术前单用化疗方案在有效性上显著改善，在安全性上与普通的术前化疗方案相比并无显著差异。

这一研究是全球首个取得成功的肺癌免疫新辅助治疗Ⅲ期研究，对NSCLC的新辅助免疫治疗方案制定有着重要意义。同时，该研究如5年OS、PFS等长期的疗效数据值得进一步期待，有待未来的长期随访研究。

## 2 NSCLC新辅助免疫治疗的获益人群

根据上述对新辅助免疫治疗的有效性和安全性相关研究，可以确定的是对Ⅰa期-Ⅲa期和Ⅲb（T3N2）期，即可切除的NSCLC患者，无论使用新辅助免疫治疗联合含铂的双药化疗、新辅助单药免疫治疗或双ICIs（O+Y）新辅助免疫治疗的方案，较术前单纯化疗方案相比均是更为有效的治疗方案，且不存在额外的安全性风险。大部分研究选择的人群为Ⅰb期-Ⅲb（T3N2）期，未包含Ia期，这可能与Ia期较少行术前化疗的原因相同，是因为Ia期肿瘤在临床上治疗方案多选择单纯手术切除，通常不需要额外进行可能会延迟手术时间的术前新辅助治疗。不可忽视的是，这些试验大都要求排除了*EGFR*突变及*ALK*重排的NSCLC患者，这是因为CheckMate-57、KEYNOTE-010等多项研究表明对存在*EGFR*突变及*ALK*重排的NSCLC患者，无论是用免疫治疗方案取代目前的化疗和靶向治疗方案，还是在化疗和靶向治疗的基础上追加免疫治疗方案，在各项生存指标和病理缓解率上均未取得明显的获益，且毒副作用的发生率有所上升。因此，在*EGFR*突变及*ALK*重排的NSCLC患者适用靶向治疗的同时不适用联合免疫治疗方案已成为该类治疗的共识^[14, 15]^。

## 3 NSCLC新辅助免疫治疗的治疗周期

术前新辅助免疫治疗最早起源于黑色素瘤的治疗，其核心目标是降低手术分期，最终提高切除率或为原来不能切除的部位争取手术机会。然而，过长的治疗时间同时意味着手术时间的推迟，可能会导致肿瘤进一步进展；而过短的治疗时间会影响免疫治疗的疗效，在未降期的时候进行手术也会导致无法达到提高切除率的目标。因此，进行免疫治疗的时间和手术时机的选择在这一方案中是两个较大的难题。在2021年国际新辅助黑色素瘤联合会对6项采用抗PD-1的免疫治疗或BRAF/MEK靶向治疗的共192例黑色素瘤患者的综合分析中，新辅助免疫治疗被推荐使用6周-8周。

在NSCLC相关的新辅助免疫治疗的研究中，如上文所述大多均为Ⅰ期、Ⅱ期研究，且采用了多重治疗方案。新辅助单药免疫治疗相关的4个实验CheckMate-159、LCMC3、TOP1501和ChiCTR-OIC-17013726分别运用了4种PD-1/PD-L1免疫抑制剂，均选择了进行2个周期的治疗；新辅助免疫治疗联合化疗的实验NADIM、NCT02716038、CheckMate-816等一般选择3个治疗周期，少部分实验最长可达4个周期；双ICIs新辅助免疫治疗的实验NEOSTAR选择了3个治疗周期。在每个周期时间选择上，CheckMate-159试验选择每2周（14 d）作为1个周期，其他大部分Ⅱ期、Ⅲ期试验均选择3周（21 d）作为1个周期。综合上述实验，可以发现在NSCLC治疗中采用新辅助免疫治疗一般需要2个-4个周期，每个周期通常为3周，即通常需要42 d-84 d的治疗时间，联合治疗的周期会偏长。同时由于此类治疗在NSCLC治疗中的作用尚未完全阐明，根据方案时间，建议每2个周期进行1次复查。

## 4 NSCLC新辅助免疫治疗疗效预测

根据2020年12月发布的专家共识，新辅助免疫治疗没有明确的疗效预测标志物，用药时也无须基于这些因素，但*EGFR*突变及*ALK*重排等疗效负性因素出现时需谨慎用药^[45]^。

目前，仍未发现可以有效预测新辅助免疫治疗疗效的生物标志物。部分实验表明PD-L1表达水平和肿瘤突变负荷（tumor mutation burden, TMB）两种在免疫治疗中较为常见的疗效预测标志物在新辅助免疫治疗中同样具有一定程度的预测价值，但关系尚不明确，且这两项标志物均存在各自的问题。此外，循环肿瘤基因（circulating tumor DNA，ctDNA）、肠道菌群也被证明有预测新辅助免疫治疗疗效的价值，但同样未获得广泛的应用。

### 4.1 免疫治疗中常见的疗效预测标志物

免疫治疗的相关研究中较为常见的几种疗效预测生物标志物包括PD-L1表达水平、TMB和微卫星不稳定性（microsatellite instability, MSI）。高水平MSI（MSI-high, MSI-H）是FDA批准使用的多种肿瘤类型（主要用于结直肠癌）的ICIs筛选标志物之一，但在NSCLC相关的多项研究中发生率极低（< 1%）^[45, 46]^，难以用于NSCLC免疫相关的疗效预测。

在PD-L1表达水平和TMB这两个标志物上，不同的研究得到了不同的结果。NEOSTAR研究表明应用新辅助纳武利尤单抗单药治疗或联合化疗，MPR与治疗前更高的PD-L1水平相关^[38]^；ChiCTR-OIC-17013726研究表明PD-L1表达与MPR相关，同时TMB≥10个突变/Mb的患者EFS显著改善（HR=0.125, 95%CI: 0.02-1.03, *P*=0.022, 2）；CheckMate-159研究结果显示在Ib期-Ⅲa期的NSCLC患者中应用纳武利尤单抗单药治疗，TMB与疗效、MPR有相关性^[24]^。

然而，LCMC3研究^[23]^显示在新辅助阿替利珠单抗单药方案中，PD-L1与MPR相关，TMB与MPR没有明显相关性；NADIM研究显示纳武利尤单抗联合卡铂+紫杉醇化疗的方案中，PD-L1和TMB与MPR均无相关性，但两者与pCR具有相关性。

鉴于上述实验中分别应用了PD-1抑制剂纳武利尤单抗、信迪利单抗和PD-L1抑制剂阿替利珠单抗，或可猜想因为使用药物种类不同影响了PD-L1水平、TMB等对疗效的预测价值，但其中的作用机制尚不明确。同时，作为术前治疗的标志物，PD-L1水平和TMB在实际应用上存在一些问题：PD-L1水平因不同实验平台应用标本的新鲜度有差异、不同抑制剂检测的检测试剂也不相同等原因，导致不同实验的研究结果很难相互比较；TMB水平也存在价格高昂、检测时间长、成功率低、临界值不明等较多不足，且组织肿瘤突变负荷（Tissue tumor mutational burden, tTMB）与血液肿瘤突变负荷（Blood tumor mutational burden, bTMB）的优劣尚存在争议。由于作用机制的不明确和实际存在的问题，PD-L1水平和TMB均未成为新辅助免疫治疗中广泛选择的预测标志物。

### 4.2 新近发现的疗效预测标志物

除上述常见的生物标志物外，ctDNA及肠道菌群已被广泛证实可用于预测免疫治疗疗效。

#### 4.2.1 ctDNA

ctDNA是一种无细胞状态的胞外DNA，广泛存在于血液、滑膜液和脑脊液等体液中，是一种极具前景的新兴肿瘤标志物。在对NSCLC患者的免疫治疗中，近年已有多项试验^[47-52]^证明ctDNA的水平及清除率可以作为疗效预测标志物，用于确定需要行ICIs治疗的患者。

在NSCLC患者的免疫治疗中，近年的多项研究^[49, 50, 53]^结果显示，ctDNA水平及清除率与OS、PFS、无复发生存期（Recurrence free survival, RFS）等重要临床终点相关，当ctDNA水平下降、清除率上升时，临床预后会有明显的改善。同时，多项研究^[51, 52]^结果显示较低的ctDNA的水平与较高的部分病理缓解率（partial pathologic response, PPR）相关。这表明，ctDNA可以为接受ICIs的NSCLC患者提供准确、无创和早期的最终预后预测。

在2021年4月公布的CheckMate-816实验数据中，研究者收集了一部分纳武利尤单抗联合化疗组和化疗组3个疗程的血液，进行ctDNA的检测。结果显示，纳武利尤单抗联合组和化疗组ctDNA清除率分别为56%和34%。研究者进一步按是否清除ctDNA进行筛选后研究，发现在ctDNA清除组中，纳武利尤单抗联合组和化疗组pCR率分别为46%和13%，明显高于未筛选时的pCR率24%和2.2%。这一研究^[44]^结果再次表明，ctDNA清除率与pCR高度相关，可用于新辅助免疫治疗疗效的疗效预测。

综合上述研究，ctDNA在新辅助免疫治疗中作为疗效预测标志物具有广泛的应用前景和研究价值。然而，早期疾病患者检测ctDNA较为困难，这在一定程度上限制了其作用^[48]^。

#### 4.2.2 肠道菌群

肠道菌群是居住在人体肠道内的正常微生物群体。近年来，许多证据^[54-59]^表明驻留在肠道内的细菌的组成可能在多种肿瘤免疫治疗的疗效方面起着关键作用，肠道菌群多样性水平高的患者应用ICIs治疗的疗效有较大幅度的提升，而部分菌群失调和治疗前应用抗生素的患者应用ICIs的治疗效果较差。因此，许多研究着眼于通过肠道菌群多样性水平的高低，确定肿瘤患者应用免疫治疗的疗效。

在NSCLC的免疫治疗上，有研究^[59]^表明在转移性NSCLC患者中，抗生素和抗PD-1/PD-L1治疗会改变了肠道菌群，抑制了肿瘤对ICIs的反应。此外，有多项针对中国人群的研究^[54, 55]^表明，接受ICIs治疗的晚期NSCLC患者中，肠道微生物多样性与抗PD-1免疫治疗的疗效有很强的相关性，肠道微生物多样性水平高的患者较水平低的患者有显著的PFS延长。这些研究表明，肠道菌群组成与NSCLC对ICIs的反应性密切相关，可以作为免疫治疗的疗效预测标志物。

目前对新辅助免疫治疗与肠道菌群之间联系的研究相对较少。在2021年5月发表的一项术前应用纳武利尤单抗联伊匹单抗新辅助免疫治疗的研究^[60]^中，肠道菌群被证实会影响该方案的疗效。

### 4.3 基因突变对新辅助免疫治疗疗效的影响

在免疫治疗方案中，如前文所述，在*EGFR*突变及*ALK*重排的NSCLC患者适用靶向治疗的同时不适用联合免疫治疗方案已成为该类治疗的共识^[14, 15]^，大部分免疫治疗相关的研究也均在选择样本时排除了*EGFR*突变及*ALK*重排。而对于*STK11*突变，目前的研究结论有差异，MYSTIC研究^[61]^显示*STK11*突变的NSCLC患者对度伐利尤单抗预后差，而KEYNOTE-042研究^[17]^则认为*STK11*突变与接受帕博利珠单抗的NSCLC患者的预后没有明显关联，故对*STK11*突变的作用尚不明确。而在与新辅助治疗相关的研究^[33]^中，NADIM研究结果显示*STK11*、*Keap1*、*RB1*和*EGFR*突变等存在与应用3个周期纳武利尤单抗联合紫杉醇+卡铂这一新辅助免疫治疗方案的NSCLC患者的中位PFS相关。

## 5 NSCLC新辅助免疫治疗亟待解决的问题和进一步研究的方向

近年来免疫治疗的迅速发展已彻底改变了多种癌症的治疗方式。新辅助免疫治疗在众多研究中展现出良好的临床应用前景，部分新近研究对新的ICIs应用于新辅助治疗提供了大量的数据支持。然而，目前该方面研究多为Ⅰ期、Ⅱ期研究，数据量少而误差较大。而在Ⅲ期研究数据方面，CheckMate-816研究已经在术前纳武利尤单抗+含铂药物化疗这一方案上取得了重大进展，但因为其他类型ICI的Ⅲ期研究均尚在进行中，未公布实验数据。对各类ICI和使用周期的选择尚需待其他Ⅲ期实验公布实验数据。

在新辅助免疫治疗的获益人群上，目前的研究结果支持对肺癌指南中可行手术切除的Ib期-Ⅲa期及Ⅲb（T3N2）期NSCLC患者，在无*EGFR*突变及*ALK*重排等特殊基因突变的情况下应用新辅助免疫治疗方案。而在具体的方案选择中，单药新辅助免疫治疗方案通常需2个周期，每个周期3周左右，即通常需要42 d左右的治疗时间；而联合化疗及其他ICIs则通常需3个周期，少部分研究最长可达4个周期，即通常需要63 d左右的治疗时间，最长可达84 d。尽管众多实验就治疗周期进行了同样的选择，尚无直接的对照实验确定各种治疗方案的具体周期。

在方案选择上，新辅助免疫治疗联合化疗的方案有着最高的有效性，但较单药治疗毒性较重、安全性略低，且治疗周期有较明显的延长趋势，建议结合临床手术时间、患者耐受情况等实际情况决定选择单药或是联合方案。同时，免疫联合化疗的相关机制尚存在不明确的地方，二者之间具有协同作用还是单纯作为两种治疗方案的叠加尚无法回答。

在疗效预测上，无论是过去常用的免疫治疗疗效预测指标，还是新近发现的指标，均存在不同程度的问题和使用限制，尚无一类可以广泛应用到临床实践的疗效预测标志物，这将使得临床上判断患者是否适用该类型治疗方案的难度较大。据此可以预测，在今后一段时间内寻找合适的疗效预测标志物仍然将是新辅助免疫治疗的重点研究方向。
